# Chemical Composition and Nutritional Value of Flowers and Fruits of *Cytisus striatus* (Hill) Rothm

**DOI:** 10.3390/plants13152121

**Published:** 2024-07-31

**Authors:** Débora Caramelo, Inês Pitacas, Cláudia Vitória, Joana Gonçalves, Jorge Gominho, Eugenia Gallardo, Ofélia Anjos

**Affiliations:** 1 CEF, Centro de Estudos Florestais, Laboratório Associado TERRA, Instituto Superior de Agronomia, Universidade de Lisboa, 1349-017 Lisboa, Portugal; dbrcaramelo@gmail.com (D.C.); jgominho@isa.ulisboa.pt (J.G.); 2 CERNAS-IPCB, Centre for Natural Resources, Environment and Society, Instituto Politécnico de Castelo Branco, 6001-909 Castelo Branco, Portugal; inespitacas@ipcb.pt (I.P.); cvitoria@ipcb.pt (C.V.); 3 Centro de Investigação em Ciências da Saúde (CICS-UBI), Universidade da Beira Interior, 6200-506 Covilhã, Portugal; joanadgoncalves13@gmail.com (J.G.); egallardo@fcsaude.ubi.pt (E.G.); 4 Laboratório de Fármaco-Toxicologia—UBIMedical, Universidade da Beira Interior, 6200-506 Covilhã, Portugal; 5 CBP-BI, Centro de Biotecnologia de Plantas da Beira Interior, 6001-909 Castelo Branco, Portugal

**Keywords:** Faboideae, *Cytisus*, nutritional value, minerals, cytotoxicity

## Abstract

In ancient times, the shoots of certain species within the *Cytisus* genus were used as animal feed. *Cytisus striatus* is a plentiful and widespread shrub that has long been utilized as a soil fertilizer in the Iberian Peninsula. The flowers of this shrub have traditionally been employed for medicinal purposes. However, the nutritional value of yellow broom flowers and fruits remains largely unexplored. In this study, flowers and fruit of *C. striatus* (*Cytisus striatus*) were collected from natural shrubs at three different locations in Portugal during the same year. An analytical assessment of their macro and micronutrient content was conducted. Regarding nutritional composition, flowers and fruits exhibited a fibre content of 18% and 42%, protein content of 21% and 12%, lipid content of 2% and 1%, carbohydrate content of 43% and 14%, and ash content of 4% and 3%, respectively. Potassium was the most abundant mineral, with concentrations of approximately 20,094 mg/kg in the flowers and 11,746 mg/kg in the fruits, followed by calcium, phosphorus, and magnesium. Compared to some edible flowers and fruits, these plant parts of *C. striatus* showed macro and micronutrient values similar to species such as lavender, lupins, and cowpea pod husks.

## 1. Introduction

In the forest typology chart for mainland Portugal, 4.3% of the forest area is occupied by brooms (species of the *Cytisus* genus), which are known as “*giestal*” and correspond to around 14,500 ha. The “*giestal*” area (90%) occurs mainly in biogeographical units located north of the Tejo river, in more inland regions [1]. Over the years, these species have expanded their territory, forming dense bushes due to their rapid growth and good adaptation to the climate. Some of them have attracted particular attention from researchers in the context of possible applications for phytoremediation processes, as they are characterised by using atmospheric nitrogen to fertilise the soils in which they are located [2]. Additionally, there is evidence that *Cytisus multiflorus* and *Cytisus scoparius* have been used as food for goats and sheep in times of nutrient scarcity.

*Cytisus striatus* (Hill) Rothm. of the Fabaceae family and Faboideae subfamily is a shrub commonly known as Portuguese broom and is considered a native species in the Iberian Peninsula and northern Morocco [3]. Given its anti-inflammatory properties, it has been widely used to make animal bedding and in folk medicine. For example, infusions of *C. striatus* (*Cytisus striatus*) flowers have been used for gout and rheumatic disorders, hypotension, heart failure, joint and muscle pain, and liver failure [4]. However, unlike the other species, *C. striatus* has been little researched. Its uncontrolled spread can present problems regarding fire propagation and wildlife accessibility. In addition, *C. striatus* has been considered an aggressive invasive species by the CalEPPC [5]. Furthermore, unlike the other species of the *Cytisus* genus, this one has not been subjected to extensive research.

Currently, according to a study by Poore and Nemecek [6], livestock production occupies a large proportion of agricultural land worldwide, but only a small percentage of livestock production relates to protein production. This issue has led to a search for new protein sources, particularly plant-based ones, as the world’s population increases and meat consumption consequently rises [7,8,9]. One example studied as a possible substitute for meat protein is *Lupinus albus* (lupin), not to mention the products that have already been developed using soya beans [10,11,12,13].

Considering all these factors, our study aimed to analyse the nutritional potential of the flowers and fruits of the Portuguese broom, harvested in three different locations in Portugal. An analysis of protein, fat, and fibre was carried out, as well as an analysis of micronutrients. In addition, cytotoxicity tests were conducted to ascertain whether or not the plant material could be beneficial to health. To our knowledge, this is the first study to be carried out on the nutritional and elemental quantification of the flowers and fruits of this species native to the Iberian Peninsula. This species can serve as an example that, nowadays, we must search for new potential resources in our surroundings to contest negative environmental impacts.

## 2. Results and Discussion

### 2.1. Cytotoxicity Analysis

**Figure 1:**
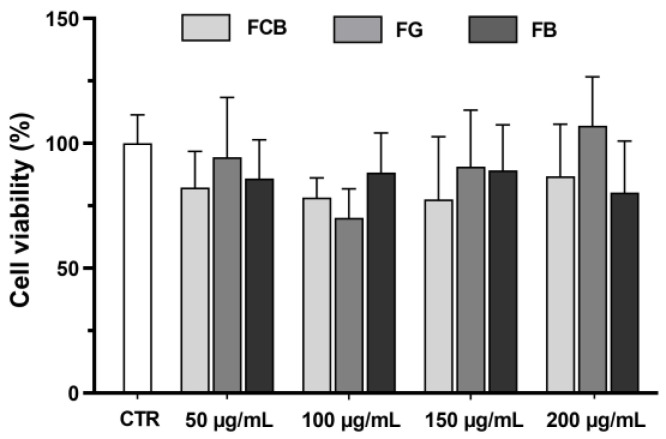
Effect of *C. striatus* (*Cytisus striatus*) flower extracts (FCB: flowers of Castelo Branco, FG: flowers of Guarda, FB: flowers of Bragança) on the viability of Caco-2 cells (MTT assay). Results are expressed as fold variation relative to the untreated control group. Error bars indicate mean ± S.E.M. No significant difference was observed when performing ANOVA followed by Dunnett’s multiple comparison test.

**Figure 2:**
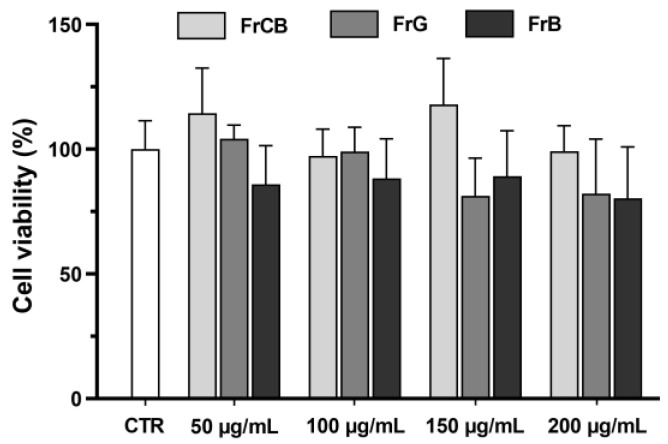
Effect of *C. striatus* fruit extracts (FrCB: fruits of Castelo Branco, FrG: fruits of Guarda, FrB: fruits of Bragança) on the viability of Caco-2 cells (MTT assay). Results are expressed as fold variation relative to the untreated control group. Error bars indicate mean ± S.E.M. No significant difference was observed when performing ANOVA followed by Dunnett’s multiple comparison test.

One of the main objectives of this study was to analyse the cytotoxicity of *C. striatus* flowers and fruit using ethanolic extracts. The results of the MTT assay, presented in Figure 1, showed that the flower extracts from the three different sites were not toxic at the different concentrations studied. The flowers of this species averaged 89.0% ± 18.3% viability at 50 µg/mL, 92.9% ± 27.0% at 100 µg/mL, 98.0% ± 29.9% at 150 µg/mL, and 102.8% ± 21.6% at 200 µg/mL, considering the three sites. Although there was a slight increase in cell viability in Guarda flower extracts from 100 µg/mL to 200 µg/mL, this result was not statistically significant. The data for the fruits are given in Figure 2, and these were also considered non-toxic, showing averages of 101.4% ± 18.1%, 95.7% ± 11.7%, 100.8% ± 21.0%, and 91.4% ± 16.9% for the concentrations of 50 µg/mL, 100 µg/mL, 150 µg/mL, and 200 µg/mL, respectively. It is possible to observe that some samples showed cell viability above 100%, particularly those from Castelo Branco. However, as the statistical analysis showed no significant differences, these values are not unreasonable.

In contrast to our results, a study conducted by Bouziane et al. [14] analysed aqueous and ethyl acetate extracts of the aerial parts of *Cytisus villosus*. The cytotoxicity of these extracts was evaluated using the MTT assay on two human breast cancer cell lines (T47D, MCF7) and one colon cancer cell line (HCT116). In the aforementioned study, the results indicated that both extracts inhibited the growth of the human cancer cell lines. The ethyl acetate extract showed median lethal dose 50 (LD50) values between 1.57 and 3.2 mg/mL, while the aqueous extract values ranged between 2.6 and 5.4 mg/mL. These findings may be attributed to the different species under study. It is also important to note that additional cytotoxicity tests should be conducted using other cell lines.

### 2.2. Nutritional Parameters of Flowers and Fruits

Table 1 presents the results based on dry matter (DM), showing that the flowers of *C. striatus* from three different locations have protein values ranging from 18.70% to 21.62% and fat content ranging from 1.65% to 2.29%. The crude fibre content ranges from 17.32% to 19.97%, while the ash content varies between 3.95% and 5.23%. Additionally, the DM content of the samples ranges from 86.82% to 89.40%.

In contrast, the protein content of the fruits averages 11.58%, which is lower than that of the flowers. The fruits have an average DM content of 92.96%, ash content of 2.86%, and fat content of 1.05%, while the fibre content reaches 41.93%. It should be noted that the lignin content (11.33% to 12.34%), NDF (neutral detergent fibre), and ADF (acid detergent fibre) values are higher in the fruits than in the flowers, likely due to the inclusion of seeds and their coatings in the analysis.

The analysis of variance for nutritional parameters revealed significant differences (*p* < 0.0015) among various parts of the plant and harvest locations, with values ranging from 0.1 for fibre NDF to 33.1 for ash. However, cellulose showed no significant differences with respect to the interaction between locality and plant part (LxP), and lignin content did not vary significantly with respect to locality (L).

Regarding micronutrients (Table 2), it was found that the flowers and fruit contained considerable concentrations of Ca, Mg, Mn, and P, with K being the mineral with the highest concentration, ranging between 16,349.4 mg/kg and 25,872.5 mg/kg for flowers and 10,310.0 mg/kg and 12,950.9 mg/kg for fruit, respectively. Generally, the fruit had lower micronutrient levels compared to the flowers, except for Na. Furthermore, Ca and Mn levels exhibited variations between sites, with FB and FrB showing higher values than the other sites. The statistical analysis indicated that Ca and P were the only minerals that did not show significant differences in relation to the interaction, while the others showed a variable range of differences, such as Fe (5.2), and some showed more pronounced ones, such as Na (33.1). In particular, Cu was the only mineral that exhibited a marginal significance (0.01 < *p* < 0.05) in relation to the plant part and LxP variation, with no significant result observed for harvest location. This result was not observed for the other minerals, which showed a statistically significant difference (*p* > 0.001) in relation to the analysed variance factors.

Pinela et al. [15] analysed flowers from four species of shrubs belonging to the same tribe (*Genisteae*), including *C. striatus*, using freeze-drying and room-temperature drying methods. Their study revealed protein values of 13.96% and 21.04% for shade-dried and freeze-dried flowers, respectively. Our results indicate protein levels similar to the freeze-dried flowers and higher than those of the shade-dried flowers in their study, although with lower fat content and comparable ash values. This difference in protein content could be attributed to varying harvest times and subsequent climatic conditions. Grzeszczuk et al. [16] assessed the nutritional values of edible flowers such as *Salvia splendens* (scarlet sage), *Bellis perennis* L. (white daisy), *Viola tricolor* L. (heartsease), and *Lavandula angustifolia* Mill. (lavender), finding protein contents ranging from 12% to 80% by dry weight in different species. Our study found that *C. striatus* flowers had a higher protein content compared to lavender and heartsease, but lower than white daisy and scarlet sage. The fibre content of *C. striatus* flowers is similar to that of *Lavandula angustifolia* and exceeds that of the other species listed. Fernandes et al. [17] conducted a review in which most of the examined edible flowers exhibited substantial potassium (K) values, ranging from 13,000 mg/kg to 40,600 mg/kg dry weight for *Begonia boliviensis* and *Tagetes patula*, respectively. *C. striatus* flowers displayed values within these ranges for most minerals, with magnesium (Mg) levels slightly surpassing those of the highest species in their study. Additionally, manganese (Mn) content in *C. striatus* flowers exceeded that of *Brassica oleracea var. italica* (351 mg/kg). In contrast, compared to the fruits of *Arbutus unedo* (widely used in brandy production in Portugal), broom fruits exhibited higher levels of protein, K, Mn, and phosphorus (P), along with a lower fat content [18].

Sujak et al. [19] investigated the nutritional profiles of lupin seeds from three species, finding higher protein values (between 33% and 47%) compared to broom fruits, while the fibre content was significantly lower (approximately 14%). On the other hand, the bitter seeds of four species of lupin [20] had a notably higher fat content (ranging from 5.15% to 16.12% in DM) compared to broom fruits (on average 1%). A review of elemental analysis outlined the mineral composition of white lupin seeds, indicating values of Ca ranging from 2100 to 4700 mg/kg, P from 4300 to 7200 mg/kg, Mg from 1200 to 2200 mg/kg, and K from 8600 to 11,100 mg/kg [21]. The Na content was between 100 and 200 mg/kg, while the Mn content averaged around 896 mg/kg. Lupin seeds of various species also have remarkably high protein content. However, regarding micronutrients, the values obtained from broom fruits align with those of *Lupinus albus* seeds for Ca, Mg, K, and Na.

Since our analysis included the seed and its covering (the whole pod), comparing pods from species similar to *C. striatus* is essential. Comparing similar species, Abebe et al. [22] verified the protein and fibre content of cowpea (*Vigna unguiculata* L.) pod husks (12.7% and 31.8% in DM, respectively), while whole cowpeas had a protein content of 16–31%, and raw cowpea seeds ranged from 23.56% to 26.14%. Gerrano et al. [23] examined immature cowpea pods to assess the variation in mineral elements and total protein. Although broom fruits exhibit similar profiles to cowpea pod husks, the state of ripeness can influence the results, as observed in the comparisons in our study.

According to the daily reference intakes (DRIs) [24,25] for minerals for adults, we can verify that the flowers and fruits of *C. striatus*, if considered as food, are high in K (twice above 300 mg/100 g), Ca (>120 mg/100 g), Mg (>56.25 mg/100 g), Fe (>2.1 mg/100 g), Cu (>0.15 mg/100 g), and Mn (>0.3 mg/100 g). In the case of Zn (>1.5 mg/100 g) and P (>105 mg/100 g), the flowers have a high content, and the fruit has a significant amount. The Na content in flowers and fruit is below 0.04 g/100 g, which means they have very low amounts of salt as food. These results are relevant because elements such as potassium contribute to protein synthesis, calcium strengthens bone structure, magnesium aids the metabolic functioning of cells, and iron is essential for the transport of oxygen to cells. Copper plays an important role in the production of haemoglobin, and manganese is responsible for activating many enzymes [17,26].

In terms of reference intakes for nutrients, the flowers are high in protein and fibre, and low in fat. The fruits are a source of protein, high in fibre, and low in fat.

### 2.3. Multidimensional Approach

**Figure 3:**
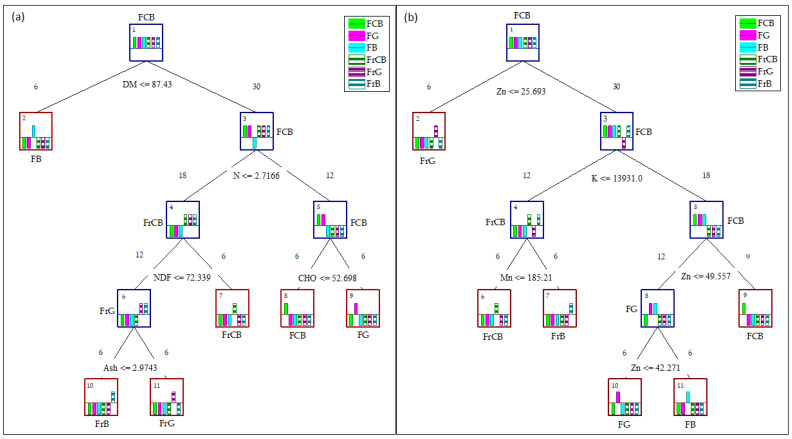
Results of the decision tree for discriminating parameters of the flowers and fruits of *C. striatus* with the most influential nutritional (**a**) and mineral parameters (**b**). Decision nodes are depicted in blue, and terminal nodes in red.

Figure 3 shows a decision tree constructed based on nutritional parameters and mineral content using the Gini algorithm. Each case in this study consisted of six replicates, and notably, all nodes effectively segregated the samples within each group accurately. Separation based on nutritional values is primarily influenced by variables such as DM, N, NDF, CHO, and ash content (Figure 3a). In contrast, separations based on mineral content predominantly identify Zn, K, and Mn as significant discriminators. In Figure 3b, Zn content mainly distinguishes FCB from the other samples, while K content separates FrCB from FrB, with Mn concentration further distinguishing these groups. Notably, Zn plays a significant role in discriminating between flowers from different locations, and also serves as a distinguishing factor between fruits and flowers of *C. striatus*, along with K.

**Figure 4:**
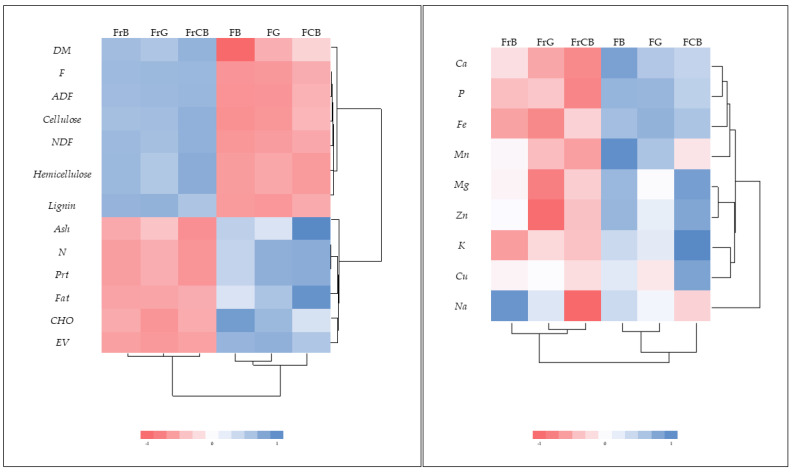
Heat maps depicting clusters of nutritional values and mineral contents of the flowers and fruits of *C. striatus*. Abbreviations used: DM, dry matter; N, nitrogen content; Prt, protein; Fat, crude fat; F, crude fibre; CHO, carbohydrate; EV, energetic value; NDF, neutral detergent fibre; ADF, acid detergent fibre; NFC, non-fibrous carbohydrate; Ca, calcium; Mg, magnesium; K, potassium; Na, sodium; Fe, iron; Cu, copper; Zn, zinc; Mn, manganese; P, phosphorus. Labels: FCB, flowers of Castelo Branco; FG, flowers of Guarda; FB, flowers of Bragança; FrCB, fruits of Castelo Branco; FrG, fruits of Guarda; FrB, fruits of Bragança.

To better visualize how the cases and variables are grouped, a heat map was generated (Figure 4). The heat maps grouped the flowers and fruits of the broom into two clusters based on the abundance of nutritional values and minerals. Visibly, two clusters were obtained by distinguishing between flowers and fruits: the first cluster consisted of the parameters DM, F, ADF, NDF, cellulose, hemicellulose, and lignin, indicating that the fruit has higher fibre content than the flowers. The second cluster, formed by the content of proteins, fats, carbohydrates, energy value, and ash, shows that the flowers have higher levels of these parameters compared to the fruit. Regarding minerals, their content levels differed between flowers and fruits, with the latter showing lower levels of Ca, P, Fe, Mn, Mg, Zn, K, and Cu. Additionally, it is possible to observe the variation in Na content between fruits and flowers at different sites, as mentioned above.

## 3. Materials and Methods

### 3.1. Sample Preparation

**Figure 5:**
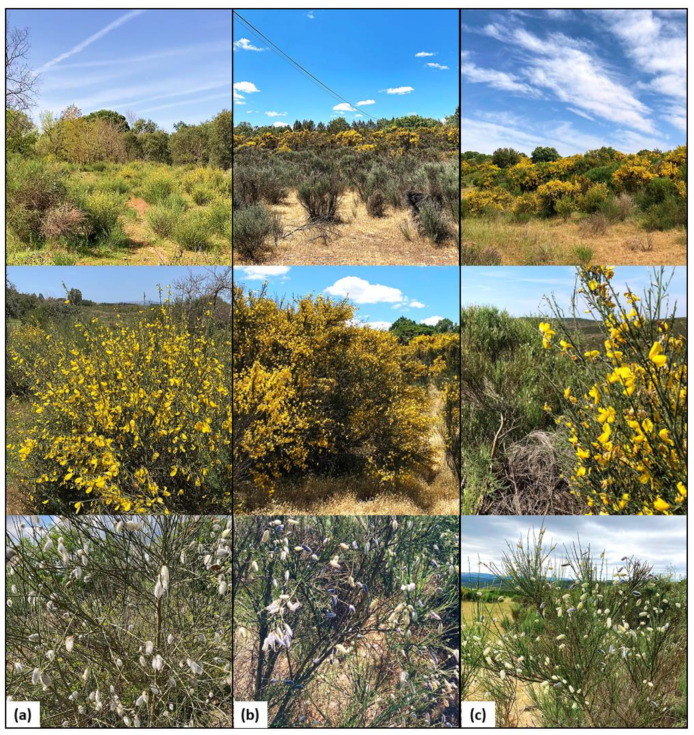
Habitat, flowers, and fruit (from top to bottom) collected at the different study locations: Escola Superior Agrária, Castelo Branco (**a**); Vila Fernando, Guarda (**b**); and Montesinho Natural Park, Bragança (**c**).

The flowers and fruit samples were collected from three different locations: Escola Superior Agrária in Castelo Branco (39°49′36.2″ N 7°27′44.0″ W), Vila Fernando in Guarda (40°29′27.5″ N 7°10′04.6″ W), and Montesinho Natural Park in Bragança (41°56′13.1″ N 6°37′46.4″ W) (Figure 5). Table 3 presents a comparative analysis of the morphological characteristics of the flowers and fruit harvested at different sites. The flowers were dried at room temperature for 7 days, and the fruits were dried under the same conditions for 14 days. Subsequently, the samples were ground using a Moulinex shredder and stored in a dark place until analysis. To calculate the moisture content of the samples, Formula (1) was used to determine the initial moisture content (H1). Subsequently, 2.5 g of each sample was dried at 105 ± 2 °C until a constant weight was achieved (approximately 5–6 h) in a forced draft oven (Memmert UL 60, Memmert GmbH, Schwabach, Germany). This allowed for the determination of the second moisture content (H2) using Formula (2).

The formulas used were as follows:

H1 (%) = (W − W′)/W × 100(1)

H2 (%) = (WS − W″)/WS × 100(2)

where:

- W represents the weight of the fresh sample;
- W′ represents the weight of the sample after the first drying;
- WS represents the 2.5 g portion of the sample weighed;
- W″ represents the weight of the sample dried at 105 °C.

### 3.2. Cytotoxic Assays

5 g of each sample was extracted with 96% ethanol by maceration for 24 h on a stirring plate. The samples were then filtered and evaporated in a rotary evaporator. Subsequently, they were dissolved in ethanol to achieve a known concentration of flower extract (50 mg/mL) and fruit extract (25 mg/mL). The samples were stored in cold storage until further analysis.

The cytotoxicity of the extracts was evaluated using a 3-[4,5-dimethylthiazol-2-yl]-2,5 diphenyl tetrazolium bromide (MTT) assay. For this, a colorectal adenocarcinoma cell line (Caco-2) (database name: American Type Culture Collection (ATCC); accession number: HTB-37) was cultured in Roswell Park Memorial Institute (RPMI) medium supplemented with 1% antibiotic-antimycotic mixture and 10% foetal bovine serum (FBS). The cells were maintained at 37 °C in an atmosphere containing 5% CO_2_.

For the MTT assay, cells were seeded in 96-well plates (catalogue number 734.2802, Avantor, VWR, Amadora Portugal) at a density of 0.5 × 104 cells per well and incubated for 24 h until confluence. Once confluent, cells were exposed to different concentrations of the prepared extracts for 6 h: 50 µg/mL, 100 µg/mL, 150 µg/mL, and 200 µg/mL. RPMI medium served as a negative control. After incubation, the medium was aspirated, and MTT solution (0.5 mg/mL) was added to each well. The cells were then incubated for 3 h. Dimethyl sulfoxide (DMSO) solution was used to dissolve the formazan crystals formed, and absorbance was measured at 570 nm using the xMark™ microplate absorbance spectrophotometer (Bio-Rad Laboratories, Hercules, CA, USA). All experiments were performed in four independent assays.

### 3.3. Nutritional Analysis

According to AOAC (2000) [27], samples of *C. striatus* flowers and fruits were analysed for dry matter (DM), total ash (Ash), protein (Prt), fibre (F), and fat (Fat). The analyses of neutral detergent fibre (NDF), acid detergent fibre (ADF), and acid detergent lignin (ADL) were conducted following the procedures outlined by Van Soest et al. [28].

### 3.4. Mineral Analysis

Micronutrients were analysed by initially ashing 1 g of the samples in a muffle furnace at 480 °C for 16 h, followed by digestion with 3 mL of 20% (*v*/*v*) hydrochloric acid on a hotplate. The elements calcium (Ca), magnesium (Mg), potassium (K), manganese (Mn), copper (Cu), lead (Pb), cadmium (Cd), zinc (Zn), iron (Fe), chromium (Cr), nickel (Ni), and sodium (Na) were quantified using atomic absorption spectroscopy (iCE3000, Thermo Scientific, Waltham, MA, USA). Phosphorus (P) was quantified using molecular absorption spectrophotometry (spectrophotometer Thermo Electron Corporation evolution 300 LC, Waltham, MA, USA). For the minerals chromium (Cr), cadmium (Cd), lead (Pb), and nickel (Ni), detection limits were set at 0.04 mg/kg, 0.04 mg/kg, 1.26 mg/kg, and 1.62 mg/kg, respectively, due to the values obtained falling below these thresholds.

### 3.5. Statistical Analysis

The cytotoxic analysis data underwent one-way ANOVA with Dunnett’s multiple comparison test (*p* < 0.05 for significance) using GraphPad Prism 7 software. Factorial ANOVA was employed to assess differences in macro and micronutrient content across raw materials, considering collection site and species parts as factors, and applying the Scheffe test (*p* < 0.05) for evaluating individual mean differences. STATISTICA 7 software was utilized for ANOVA, classification trees, and heat maps.

## 4. Conclusions

To the best of our knowledge, this study represents the first evaluation of nutrients in *C. striatus* flowers and fruits from three different locations in Portugal. Generally, the flowers exhibit a composition similar to several edible flowers, containing significant levels of minerals such as potassium, calcium, magnesium, iron, copper, manganese, crude protein, and fibre. Similarly, the fruits are also rich in protein and fibre, along with the aforementioned minerals. Regarding toxicity assessed in human cells, both samples demonstrated non-toxic attributes; however, further toxicological assessments are imperative. Future research should focus on vitamin profiles and other classes of compounds to enhance the nutritional value of these plant parts. Overall, this study provides valuable information for integrating these flowers and fruits into both human and livestock diets.

## Figures and Tables

**Table 1:** Nutritional composition of *C. striatus* (*Cytisus striatus*) flowers and fruits from Castelo Branco, Guarda, and Bragança (% dry matter). Results expressed by factorial analysis of variance (ANOVA).

	Samples						Variance Origin			
	FCB	FG	FB	FrCB	FrG	FrB	Local (L)	Plant Part (P)	LxP	Error
DM (%)	89.40 ± 0.14 ^d^	88.53 ± 0.60 ^e^	86.82 ± 0.20 ^f^	93.31 ± 0.17 ^b^	92.61 ± 0.07 ^c^	92.97 ± 0.30 ^cb^	4.2 ^(^***^)^	88.8 ^(^***^)^	6.1 ^(^***^)^	0.9
Ash (% DM)	5.23 ± 0.09 ^a^	3.95 ± 0.09 ^c^	4.22 ± 0.03 ^b^	2.63 ± 0.06 ^f^	3.09 ± 0.04 ^d^	2.86 ± 0.07 ^e^	4.1 ^(^***^)^	55.4 ^(^***^)^	33.1 ^(^***^)^	7.4
N (% DM)	3.46 ± 0.03 ^d^	3.42 ± 0.03 ^d^	2.99 ± 0.04 ^c^	1.77 ± 0.04 ^f^	1.95 ± 0.10 ^e^	1.84 ± 0.10 ^fe^	1.7 ^(^***^)^	94.6 ^(^***^)^	3.2 ^(^***^)^	0.5
Prt (% DM)	21.62 ± 0.18 ^d^	21.35 ± 0.21 ^d^	18.70 ± 0.24 ^c^	11.08 ± 0.24 ^f^	12.18 ± 0.65 ^e^	11.48 ± 0.63 ^fe^	1.7 ^(^***^)^	94.6 ^(^***^)^	3.2 ^(^***^)^	0.5
Fat (% DM)	2.29 ± 0.14 ^c^	1.90 ± 0.06 ^d^	1.65 ± 0.16 ^e^	1.07 ± 0.04 ^f^	1.04 ± 0.08 ^f^	1.03 ± 0.05 ^f^	5.7 ^(^***^)^	83.2 ^(^***^)^	8.6 ^(^***^)^	2.5
F (% DM)	19.97 ± 0.31 ^e^	17.58 ± 0.44 ^f^	17.32 ± 0.27 ^f^	42.15 ± 0.83 ^d^	42.02 ± 0.35 ^d^	41.62 ± 0.58 ^d^	0.2 ^(^***^)^	96.6 ^(^***^)^	0.0 ^(^***^)^	3.1
CHO (% DM)	50.89 ± 0.26 ^d^	55.22 ± 0.54 ^c^	58.10 ± 0.55 ^b^	43.07 ± 0.64 ^e^	41.68 ± 0.68 ^f^	43.01 ± 0.70 ^e^	3.8 ^(^***^)^	87.2 ^(^***^)^	8.6 ^(^***^)^	0.5
EV (kcal)/100 g	350.56 ± 0.77 ^e^	358.55 ± 0.78 ^d^	356.73 ± 0.54 ^d^	310.52 ± 1.72 ^f^	308.79 ± 0.91 ^f^	310.46 ± 1.08 ^f^	0.3 ^(^***^)^	98.5 ^(^***^)^	1.1 ^(^***^)^	0.1
NDF (% DM)	31.40 ± 0.27 ^e^	29.20 ± 0.58 ^f^	27.93 ± 0.73 ^f^	72.78 ± 0.33 ^b^	68.39 ± 0.46 ^d^	70.14 ± 1.53 ^c^	0.4 ^(^***^)^	99.4 ^(^***^)^	0.1 ^(^***^)^	0.1
ADF (% DM)	23.20 ± 0.86 ^e^	19.91 ± 0.38 ^f^	19.58 ± 0.80 ^f^	44.91 ± 0.96 ^d^	44.36 ± 0.60 ^d^	43.95 ± 0.74 ^d^	0.5 ^(^***^)^	98.8 ^(^***^)^	0.4 ^(^***^)^	0.2
Hemicelluloses (% DM)	8.20 ± 1.03 ^f^	9.29 ± 0.61 ^f^	8.34 ± 0.26 ^f^	27.87 ± 1.03 ^d^	24.03 ± 0.57 ^e^	26.19 ± 1.40 ^d^	0.3 (**)	97.2 (***)	1.9 (***)	0.6
Cellulose (% DM)	17.84 ± 0.72 ^e^	15.18 ± 0.35 ^f^	14.76 ± 0.78 ^f^	33.59 ± 1.62 ^d^	32.02 ± 0.57 ^d^	31.72 ± 0.69 ^d^	1.2 ^(^***^)^	98.0 ^(^***^)^	NS	0.7
Lignin (% DM)	5.36 ± 0.22 ^f^	4.73 ± 0.09 ^f^	4.82 ± 0.04 ^f^	11.33 ± 0.76 ^d^	12.34 ± 0.17 ^e^	12.23 ± 0.21 ^e^	NS	97.9 ^(^***^)^	1.5 ^(^***^)^	0.6
NFC (% DM)	39.46 ± 0.30 ^d^	43.60 ± 0.59 ^c^	47.50 ± 0.86 ^b^	12.44 ± 0.55 ^f^	15.31 ± 0.61 ^e^	14.49 ± 1.82 ^fe^	1.5 ^(^***^)^	97.2 ^(^***^)^	1.1 ^(^***^)^	0.2

Data expressed as mean value ± standard deviation; DM, dry matter; N, nitrogen; Prt, protein; Fat, fat; F, fibre; CHO, carbohydrate; EV, energetic value; NDF, neutral detergent fibre; ADF, acid detergent fibre; NFC, non-fibrous carbohydrate; FCB, flowers of Castelo Branco; FG, flowers of Guarda; FB, flowers of Bragança; FrCB, fruits of Castelo Branco; FrG, fruits of Guarda; FrB, fruits of Bragança. Mean values with the same letter in a row are not statistically different. NS, not significant; * 0.01 < *p* < 0.05; ** 0.001< *p* < 0.01; *** *p* < 0.001.

**Table 2:** Composition of calcium, magnesium, potassium, sodium, iron, copper, zinc, manganese, and phosphorus in the flowers and fruits of *C. striatus* from Castelo Branco, Guarda, and Bragança (mg/kg dry matter). Results expressed by factorial analysis of variance (ANOVA).

Samples	Ca (mg/kg DM)	Mg (mg/kg DM)	K (mg/kg DM)	Na (mg/kg DM)	Fe (mg/kg DM)	Cu (mg/kg DM)	Zn (mg/kg DM)	Mn (mg/kg DM)	P (mg/kg DM)
FCB	4421.9 ± 158.4 ^d^	2870.2 ± 129.8 ^d^	25,872.5 ± 903.5 ^c^	93.7 ± 7.5 ^e^	76.0 ± 5.5 ^d^	20.6 ± 1.6 ^e^	50.9 ± 1.2 ^c^	256.3 ± 7.9 ^e^	2341.2 ± 214.6 ^e^
FG	4693.1 ± 238.2 ^d^	1648.0 ± 83.5 ^e^	16,349.4 ± 1108.7 ^d^	118.8 ± 7.0 ^ed^	81.9 ± 4.5 ^d^	18.8 ± 0.4 ^f^	37.4 ± 0.8 ^d^	627.0 ± 53.7 ^d^	2560.8 ± 150.1 ^e^
FB	5535.0 ± 487.4 ^c^	2550.8 ± 223.2 ^d^	18,060.6 ± 307.6 ^d^	144.7 ± 10.1 ^c^	78.0 ± 2.2 ^d^	19.3 ± 0.3 ^fe^	48.0 ± 1.0 ^c^	920.2 ± 61.6 ^c^	2571.7 ± 62.9 ^e^
FrCB	2368.1 ± 147.5 ^f^	1386.8 ± 107.6 ^e^	11,976.0 ± 714.1 ^fe^	48.9 ± 3.0 ^f^	50.5 ± 3.2 ^f^	18.7 ± 0.3 ^f^	29.8 ± 1.2 ^e^	81.3 ± 7.8 ^f^	1502.0 ± 81.0 ^f^
FrG	2646.0 ± 264.1 ^fe^	924.7 ± 85.0 ^f^	12,950.9 ± 1779.9 ^e^	128.3 ± 13.5 ^dc^	39.5 ± 3.8 ^f^	19.0 ± 0.1 ^fe^	22.7 ± 0.9 ^f^	155.9 ± 6.0 ^f^	1751.5 ± 163.8 ^f^
FrB	3211.5 ± 252.1 ^e^	1602.1 ± 107.8 ^e^	10,310.0 ± 1455.9 ^f^	209.9 ± 16.9 ^b^	43.2 ± 1.4 ^fe^	18.9 ± 0.2 ^f^	35.0 ± 3.4 ^d^	300.2 ± 12.3 ^e^	1718.5 ± 165.2 ^f^
Variance origin									
Local (L)	9.4 (***)	25.3 (***)	11.8 (***)	62.1 (***)	0.0 (NS)	7.5 (NS)	21.6 (***)	29.6 (***)	3.9 (**)
Plant part (P)	87.0 (***)	63.4 (***)	61.3 (***)	1.3 (***)	92.2 (***)	15.4 (*)	71.9 (***)	54.1 (***)	89.2 (***)
LxP	NS	8.3 (***)	24.1 (***)	33.1(***)	5.2 (***)	32.5 (*)	4.6 (***)	15.5 (***)	NS
Error	3.6	3.0	2.8	3.5	2.6	44.6	1.9	0.9	6.9

Data expressed as mean value ± standard deviation. Ca, calcium; Mg, magnesium; K, potassium; Na, sodium; Fe, iron; Cu, copper; Zn, zinc; Mn, manganese; P, phosphorus; FCB, flowers of Castelo Branco; FG, flowers of Guarda; FB, flowers of Bragança; FrCB, fruits of Castelo Branco; FrG, fruits of Guarda; FrB, fruits of Bragança; Mean values with the same letter in a column are not statistically different, NS, not significant; * 0.01 < *p* < 0.05; ** 0.001 < *p* < 0.01; *** *p* < 0.001.

**Table 3:** Morphological characteristics of the flowers and fruits of *Cytisus striatus* from different collection points.

Plant Morphology/Locations	CB	G	B
**Flower**			
Size	Medium	Medium	Bigger
Colour	Bright yellow	Darker yellow	Bright yellow
Number of petals	5	5	5
**Fruit**			
Size of pods	Small	Large	Larger
Hair	Long and more than others	Long	Long

## Data Availability

Data are contained within the article.
